# The Effects of Avocado Waste and Its Functional Compounds in Animal Models on Dyslipidemia Parameters

**DOI:** 10.3389/fnut.2021.637183

**Published:** 2021-02-16

**Authors:** Jessica Elizabeth Pineda-Lozano, Alma Gabriela Martínez-Moreno, Carmen Alejandrina Virgen-Carrillo

**Affiliations:** Instituto de Investigaciones en Comportamiento Alimentario y Nutrición (IICAN), Centro Universitario del Sur, Universidad de Guadalajara, Ciudad Guzmán, Mexico

**Keywords:** avocado waste, functional compounds, dyslipidemia, animal model, review

## Abstract

Ischemic heart disease and stroke are two main causes of death that have prevailed for more than 15 years. Dyslipidemia and its parameters like hypercholesterolemia, hypertriglyceridemia, increase in low-density cholesterol, and a reduction of high-density cholesterol have been related with heart disease and risk of stroke. Approaches to improve the health and specifically reduce the risk of heart disease, such as medications and dietary interventions have been effective, but there are other potential sources of biological compounds that could have an effect due to their antioxidant properties. Avocado is a commonly consumed fruit especially its pulp, while the peel, seed, and leaf are usually discarded as waste. Some researchers have reported antioxidant, hepatoprotective, gastroprotective, lipid-lowering, and hypoglycemic effects in these wastes. In this review article, we have summarized the current evidence on the effect of biological compounds from avocado waste on dyslipidemia parameters in preclinical models. Also, we have included the compound extracted and the extraction method from the selected articles.

## Introduction

Diet-related chronic diseases are considered a serious public health problem (1). The World Health Organization points out that the two main causes of death that have prevailed for more than 15 years in the world are ischemic heart disease and stroke, which have caused 15.3 million deaths (2). Hypercholesterolemia, hypertriglyceridemia, increase in low-density cholesterol (LDL), and a reduction of high-density cholesterol (HDL) are the general parameters of dyslipidemia. They have been related to complications such as cardiac damage and atherosclerosis (3–5). Also, the evidence relates dyslipidemias with heart disease and the risk of stroke (6, 7). The diagnosis of dyslipidemias corresponds to the alteration of one or more of the following parameters: total cholesterol (TC) ≥ 240 mg/dl, LDL ≥ 160 mg/dl, HDL ≤ 40 mg/dl, and triglycerides (TGs) ≥ 200 mg/dl (7). In both animals and humans, several studies have reported the effects of food compounds to improve the signs and symptoms of various chronic diseases, such as diabetes, hypertension, dyslipidemia, and oxidative stress (8–17).

Avocado (*Persea americana* Mill) is an oleaginous fruit highly distributed and consumed worldwide. This fruit is native to Central America and South America; its domestication as food dates back more than 9,000 years (18). It belongs to the Lauraceae family; the genus *Persea* includes three species: *Persea schiedeana, Persea parvifolia*, and *P. americana*. The fruit of *P. americana* (common avocado) is made up of the peel, pulp, and seed (19). The color of the shell can be light or dark green or purple; smooth; rough; and also bright or opaque (20).

Avocado is increasingly consumed, especially for its pulp, which, in addition to its flavor, contains a large number of vitamins, minerals, and especially monounsaturated fatty acids, and in lesser amounts polyunsaturated acids. While other components of avocado (peel, seed, and leaf) are usually discarded as waste, a variety of functional compounds from the waste have been identified as polyphenols, organic acids, and flavonoids (21–23). Avocado peel contains a high concentration of bioactive compounds such as phenolic acids, flavonoids, catechins, and procyanidins; nevertheless, the content of compounds will depend on the extraction methods (24, 25). Melgar et al. examined raw material of bone and lyophilized shell and carried out a hydroethanolic extraction (ethanol:water 80/20). The extracts obtained were redissolved in 80% ethanol solution for phenolic characterization (final concentration 40 mg/dl). Twenty-nine phenolic compounds, 14 flavan-3-ols (epi) derived from catechin, nine flavonoids (derivatives of quercetin, kaempferol, and isorhamnetin glucoside), and six phenolic acids such as chlorogenic and coumaric acid derivatives (25) were found.

Functional compounds from avocado waste have shown an antioxidant effect through the reduction of pro-oxidative substances (26–28): hepatoprotective, gastroprotective, lipid-lowering, and hypoglycemic effects (8, 9, 15, 29). Other studies had evaluated some of its effects *in vitro* on human colon cancer cells (30, 31), on gut health in rats (32), and on hypercholesterolemic rats (33, 34). Some others have been related to cognitive function such as improvement in learning and memory processes in subjects exposed to diets high in fat and sugars (14, 16). Also, there is importance to promote research with ecological vision. The environmental impact is a preoccupying issue, because the consumption of food generates a large amount of waste. According to the World Bank, in 2016, the world generated 2,010 million tons of waste, driven by extremely rapid urbanization and the growth of populations. It is predicted that the world will produce 3.4 billion tons of waste by 2050 (35). Derived from this, it is important to look for some alternatives for the reuse of this type of waste, which can also have a positive impact on health.

For that, the objective of this review was to identify studies that have reported the effect of avocado waste on blood lipids in animal models.

## Search Strategy

The search of information was based on the following databases: EBSCO, PubMed, Science Direct, and Springer Link. The search terms were “Avocado peel” AND combined with the next words: “lipid”/“rats”/“*in vitro*”/“triglycerides”/“cholesterol.” “Avocado seed” AND “lipid”/“rats”/“*in vitro*”/“triglycerides”/“cholesterol.” “Avocado leaf” AND “lipid”/“rats”/“*in vitro*”/“triglycerides”/“cholesterol.” The search was carried out from January to December 2020; articles published between the years 2000 and 2020 were included.

## Eligibility Criteria

Only original articles that included investigation of extracts of peel, seed, or leaf avocado and its effects on blood lipid in animal models and *in vitro* techniques were considered. The articles excluded were those that did not evaluate the effect on blood lipids.

### Data Collection

The selected articles were systematically analyzed to obtain information concerning the effect on blood lipids attributed to extracts of peel, seed, or leaf from avocado. Preferred Reporting Items for Systematic Review and Meta-Analysis Protocols (PRISMA-P) guidelines (36) were used to perform the search.

## Articles and Data

**Figure 1:**
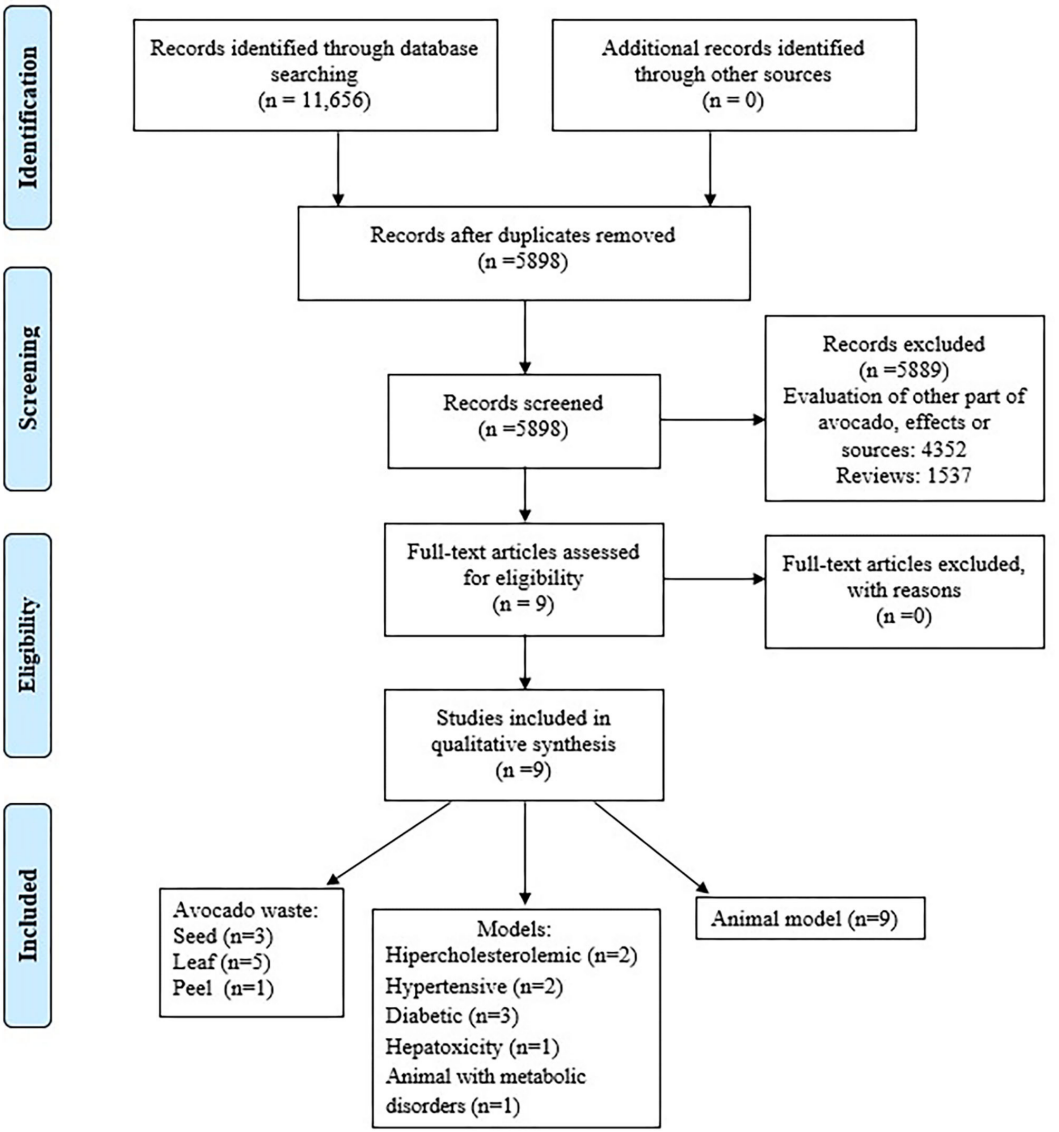
Flowchart of the election process of the articles included. Adapted from Moher et al. (36).

Through the search, we identified a total of 5,898 articles, of which 5,889 were excluded. Articles were discarded due to duplications, reviews, evaluation of different effects, and using another source. From the papers, 4,352 were excluded due to an evaluation of other compounds of avocado and other effects. Also, 1,537 were reviews. Finally, nine articles that addressed the inclusion criteria were selected. Figure 1 shows the flowchart of the election process of the articles included.

## Results

### Functional Compounds

The effects of avocado were studied in the seed (37–39), leaf (40–44), and peel (45). Of them, six used extracts to determine its effects (38, 40–44). Of the extracts, four were aqueous (40, 42–44), one was ethanolic (40), two were methanolic, (40, 42), and one was hydroalcoholic (43). Of the articles considered, just one reported the complete characterization (compound and quantity) of phenolic compounds of the avocado seed (AS), with protocatechuic acid, chlorogenic acid, syringic acid, vanillic acid, rutin, kaempferol, and kaempferide as the major phenolic compounds (37). Meanwhile, in the leaf, quercetin-3-glucoside and quercetin-3-rhamnoside were reported, but not their values (40). With respect to the peel, the following were identified (without quantities): flavanone naringenin, flavan-3-ol catechin, phenolic acid, chlorogenic acid, flavonol quercetin, cyanidin-3-glucoside, pelargonidin-3-glucoside, hydroxydelphinidin 3-glucoside, eugenol, and estragole (45) (Table 1).

**Table 1:** Phenolic method of extraction, compound content, and effect of avocado wastes used in experimental trials.

**Waste**	**Phenolic extraction**	**Compound** **μg/L**		**Time days**	**Dose per day**	**Triglycerides**	**Total cholesterol**	**LDL**	**HDL**	**Pathological model**	**References**
Avocado seed flour	Methanolic extract: methanol 75% in a Soxhlet extractor	- Protocatechuic acid - Chlorogenic acid - Syringic acid - Vanillic acid - Rutin - Kaempferol - Kaempferide	128.18 ± 0.01 0.516 ± 0.02 2.51 ± 0.002 28.67 ± 0.001 9.63 ± 0.008 2.19 ± 0.002 107 ± 0.04	6	125 mg/kg 250 mg/kg 500 mg/kg	↑ 0.020 mmol/L ↑ 0.062 mmol/L ↑ 0.078 mmol/L	↓ 35.8 mmol/L ↓ 37.7 mmol/L ↓ 39.1 mmol/L	↓ 36.3 mmol/L ↓ 36.0 mmol/L ↓ 37.7 mmol/L	↑ 0.60 mmol/L ↓ 1.50 mmol/L ↓ 1.20 mmol/L	Animal hypercholesterolemic	(37)
Ground avocado seeds extract	NS	NS	NS	21	2% of basal diet 4% of basal diet 8% of basal diet 2% of HSD 4% of HSD 8% of HSD	NR NR NR NR NR NR	↓ 0.27 g/L ↓ 0.48 g/L ↓ NS ↑ NS ↑ NS ↑ NS	NR NR NR NR NR NR	NR NR NR NR NR NR	Healthy animal model	(38)
				21	2% of basal diet 4% of basal diet 8% of basal diet	NR NR NR	↓ NS ↓ 0.27 g/L ↓ NS	NR NR NR	NR NR NR	Animal spontaneously hypertensive	
Crude seed powder	NS	- Alkaloids - Phenols - Glycosides - Flavonoids - Saponin - Gallotannin - Phalobotanin - Triterpenoids - Steroids - Resins - Anthraquinones	NS	14	SDW 500 mg/kg of ACSP DC Pioglitazone 15 mg Zolid	CV ↓ SD ↑ NS ↑ NS	CV ↓ SD ↑ NS ↑ NS	CV ↓ SD ↓ NS ↑ NS	CV ↑ SD ↑ NS ↑ NS	Animal diabetic	(39)
Aqueous extract leaf Ethanolic extract leaf Methanolic leaf extract	Aqueous Extract: Distilled water 5% w/v Ethanolic extract: Ethanol 10% w/v Methanolic extract: Methanol 10% w/v	NS	NS	28	100 mg/kg AE 100 mg/kg/day EE 100 mg/kg ME	↓ NS ↑ NS ↓ NS	↓ NS ↓ NS ↓ NS	↓ NS ↓ NS ↓ NS	↑ NS ↑ NS ↑ NS	Animal diabetic	(40)
Hydroalcoholic leaf extract	Hydroalcoholic extract: Ethanol 50% v/v	- Quercetin-3-glucoside - Quercetin-3-rhamnoside	NS	28	0.30 g/kg	↑ 24.23 mg/dl	↑ 37.79 mg/dl	NR	NR	Animal diabetic	(41)
Aqueous extract leaf Methanolic extract leaf	Aqueous extract: NS Methanolic extract: NS	NS	NS	56	10 mg/kg AE 10 mg/kg ME	NR NR	↓ 8% ↓ 5%	↓ 19% ↓ 20%	NR NR	Animal hypercholesterolemic	(42)
Aqueous extract of *Persea* *americana* leaf	Aqueous extract: distilled water	NS	NS	35	50 mg/kg 100 mg/kg 150 mg/kg	↓ 166.35 mg/dl ↓ 188.57 mg/dl ↓ 205.72 mg/dl	↓ 49.23 mg/dl ↓ 89.23 mg/dl ↓ 98.97 mg/dl	NR NR NR	↑ 10.48 mg/dl ↑ 18.86 mg/dl ↑ 32.52 mg/dl	Animal hypertensive	(43)
Aqueous extract leaf	NS	NS	NS	8	100 mg/kg 200 mg/kg	↓ 82.26 mg/dl ↓ 80.66 mg/dl	↓ 13.54 mg/dl ↓ 24.22 mg/dl	NR NR	NR NR	Animal with CCl_4_ hepatotoxicity	(44)
Avocado peel	Methanolic extract: 70% methanol:water	- Flavanone naringenin - Flavan-3-ol catechin - Phenolic acid - Chlorogenic acid - Flavonol quercetinAnthocyanins: - Cyanidin-3-glucoside - Pelargonidin-3-glucoside - Hydroxydelphinidin 3-glucosideVolatile compounds - Eugenol - Estragole	NS	98	SDW *ad-libitum* HSFG *ad-libitum* HSFG *ad-libitum* + 200 mg/kg AP	CV ↑ SD ↓ NS	CV ↑ SD ↓ NS	NR NR NR	CV ↓ NS ↓ SD	Animal with metabolic disorders: dyslipidemia, insulin resistance, and adipogenesis	(45)

*LDL, low-density cholesterol; HDL, high-density cholesterol; AE, aqueous extract; EE, ethanolic extract; ME, methanolic extract; NS, not specified; NR, not reported; HSD, high-sucrose diet; ACSP, avocado crude seed powder; SDW, standard diet and water; HSFG, high-sucrose fat diet group; AP, avocado peel; CV, control value; DC, diabetic control; SD, statistical difference compared with control group*.

### Pathologies

The objective of this article was to explore the effect of avocado wastes on blood lipids, despite this there were few studies in which their objective was to determine the effect of avocado or its extract specifically in those parameters. For that, we included articles in which serum lipids levels were evaluated, although their objective was to evaluate other diseases. Three of the articles were on a diabetic animal model (39–41), two were on a hypertensive animal model (38, 43), one used a healthy animal model (38), two were on a hypercholesterolemic animal model (37, 42), one was realized on an intoxicated animal model (44), and one was on animals with metabolic disorders (45). Articles that worked with *in vitro* techniques and evaluated lipid-lowering activity were not found (Table 1).

### Effect of Avocado Waste on Blood Lipids

The effect found of avocado waste on blood lipids was heterogeneous, depending on the model used for the research. In diabetic models, only one investigation reported values of TGs and cholesterol, and in both, an increase was identified (41). The others reported changes in TGs, TC, LDL, and HDL but did not specify the range values (39, 40). In the articles with the hypertensive animal model, they found a reduction of TC in both cases (38, 43), but only one investigation reported values of TG reduction and HDL increase (43).

The articles that reported values of blood lipids in a healthy animal model found different results in respect of TC. One found an increase in TC with diet enriched with cholesterol or sucrose (38). In the case of a hypercholesterolemic model, there was a reduction found on TG, TC, and LDL (37, 42) and an increase of HDL (37). Similar results were found in the case of the intoxicated animal model, with a reduction of TC and TGs (44). In the case of animals with metabolic disorders, it was found that the values between the experimental group that consumed avocado peel and the control group were similar, just that there was a variation in the HDL levels contrary to the significant increase of TG and TC in the high-sucrose fat diet (45) (Table 1).

## Discussion

Based on the results of the search, a relatively low number of research papers were found in which the objective was to evaluate the effect of avocado waste over blood lipids. There was a study that evaluated the lipid profile-lowering effect of AS flour at a dose of 125–500 mg/kg/day in a hypercholesterolemic animal model reporting major decreases by the highest doses of TC (39.1 mmol/L) and LDL (37.7 mmol/L); the lowest increase of TG was presented in the dose of 125 mg/kg/day (0.078); and at the same dose, an increase (0.60 mmol/L) of HDL (37) was shown. Similar results were found when avocado oil was used in a similar model with a dose of 450–900 mg/kg/day of virgin avocado oil (VAO) and simvastatin with a hypercholesterolemic diet. A reduction of TG and LDL was found in the group with a major concentration of VAO (900 mg/kg) and simvastatin (10 mg/kg). Also, in the doses of 450 mg/kg of VAO, there was found an increment in HDL and a significant reduction of LDL (34). Even though a reduction of TC and LDL and an increase in HDL were identified, but unlike in the effect of VAO, the TGs rose (37). An important difference is the period of time of the study: the first was carried out for 6 days, while the second lasted 28 days. It is important to mention that the value of phytosterol reported on VAO was 34 g/10,034 g, with β-sitosterol as a major phytosterol and small amounts of campesterol, stigmasterol, and α5-avenasterol. While in the AS flour, they identified protocatechuic acid of 128.18 μg/g and in other quantities vanillic acid (28.67 μg/g) and kaempferide (9.63 μg/g), among others. Two other studies were found that used AS: in the first study, TC decreased in healthy animals with doses of 2 and 4% (AS), obtaining a decrease of 0.27 and 0.48 g/L, respectively. In the healthy groups who consumed a diet high in sucrose, TC was reported to be elevated in all doses. The other study used diabetic animals, and in 500 mg/kg/day, a significant reduction of TG, TC, and LDL compared with those of the control group was found; also a significant increase of HDL *p* < 0.01 was shown, and the values demonstrated the best effect even when compared with the positive control who was treated with medication (39).

The hypertensive animal groups showed a decrease in TC, the most evident being 0.27 g/L in a 4% AS dose (38). Results show a wide difference between studies with the same raw material; this may be due to the fact that both the doses and the intervention time are varied (37–39).

In the case of the animal model with metabolic disorders, it was generally evidenced that the consumption of 200 mg/kg/day of avocado peel improved TG and TC levels; the subjects who only consumed the high-sucrose fat diet showed significant increases in those parameters (45). Other investigations utilized differences based on extracts; three of them utilized similar doses administrated in different periods of time (40, 43, 44). Brai et al. (44) informed a diminution of TC at 82.26 mg/dl in a dose of 100 mg/kg/day, while the highest diminution of TC (24.22 mg/dl) was 20 mg/kg/day. Dzeufiet et al. (43) evidenced a significant reduction of TG and TC dose dependently, showing a greater reduction in the dose of 150 mg/kg/day (TG 205.72 mg/dl and TC 98.97 mg/dl); also, the HDL evidenced an increase of 32.52 mg/dl. Kouamé et al. (40) reported non-significant differences in a reduction of TG in a dose of 100 mg/kg/day in aqueous extract and methanolic extract but an increase in the ethanolic extract. Those researchers proved its respective leaf extract in a different pathological model: with hepatotoxicity, hypertension, and diabetes, with varied temporality of 8, 35, and 28 days, respectively. Brai et al. (42) studied the effect of an aqueous leaf extract and a methanolic extract and found an important reduction of 8% of TC in the first one. Another study (41) was made with a hydroalcoholic leaf extract with 0.30 g/kg/day and showed an increase of TG (24.33 mg/dl) and TC (37.79 mg/dl).

## Conclusion

There is an important field of research in the area of organic waste. It becomes increasingly relevant due to concern for the environmental impact of the large volume of waste, in addition to the resources necessary for its degradation.

Organic waste has been shown to be an important source of bioactive compounds that can exert beneficial health effects. It is a fact that in the near future, humans must incorporate organic waste in the alimentation and not as an aliment but also as an ingredient, a complement, and even medicine, not only derived from the amount of functional elements that these wastes contain but also because it is necessary for humans to be environmentally responsible and to seek new ways to reuse at a low environmental cost and high impact on health.

In the present review, it was not possible to evaluate the relationship between waste compounds and biological parameters, since they were mostly not reported. In the same way, the doses, exposure times, and pathological models were varied. Therefore, the study of organic waste, specifically avocado, has a lot of potential, and evidence needs to be able to make more forceful comparisons, especially those that suggest a relationship between dose and effect; it is important to highlight that as far as we know, this is the first review to address this problem.

So we can conclude that more research is needed in this field of knowledge; although phytotherapy is already considered an innovative treatment with an important impact on health, the use of biodegradable waste has not yet been explored and completely studied.

## Author Contributions

JP-L: conception or design of the work, data acquisition, analysis or interpretation of work data, writing of the work, and final approval of the version for publication. AM-M: conception or design of the work, analysis or interpretation of work data, writing of the work, critical review of the content of the manuscript, and final approval of the version for publication. CV-C: analysis or interpretation of work data, critical review of the content of the manuscript, and final approval of the version for publication. All authors contributed to the article and approved the submitted version.

## Conflict of Interest

The authors declare that the research was conducted in the absence of any commercial or financial relationships that could be construed as a potential conflict of interest.
